# From Genome-Wide Association Studies to Cardiac Electrophysiology: Through the Maze of Biological Complexity

**DOI:** 10.3389/fphys.2020.00557

**Published:** 2020-05-27

**Authors:** Koen T. Scholman, Veronique M. F. Meijborg, Carolina Gálvez-Montón, Elisabeth M. Lodder, Bastiaan J. Boukens

**Affiliations:** ^1^Department of Medical Biology, Amsterdam University Medical Centers, University of Amsterdam, Amsterdam, Netherlands; ^2^Department of Experimental Cardiology, Amsterdam University Medical Centers, University of Amsterdam, Amsterdam, Netherlands; ^3^Netherlands Heart Institute, Utrecht, Netherlands; ^4^ICREC Research Program, Germans Trias i Pujol Health Science Research Institute, Badalona, Spain; ^5^CIBERCV, Instituto de Salud Carlos III, Madrid, Spain

**Keywords:** GWAS, cardiac electrophysiology, arrhythmias, gene expression, genetics

## Abstract

Genome Wide Association Studies (GWAS) have provided an enormous amount of data on genomic loci associated with cardiac electrophysiology and arrhythmias. Clinical relevance, however, remains unclear since GWAS do not provide a mechanistic explanation for this association. Determining the electrophysiological relevance of variants for arrhythmias would aid development of risk stratification models for patients with arrhythmias. In this review, we give an overview of genetic variants related to ECG intervals and arrhythmogenic pathologies and discuss how these variants may influence cardiac electrophysiology and the occurrence of arrhythmias.

## Introduction

**FIGURE 1:**
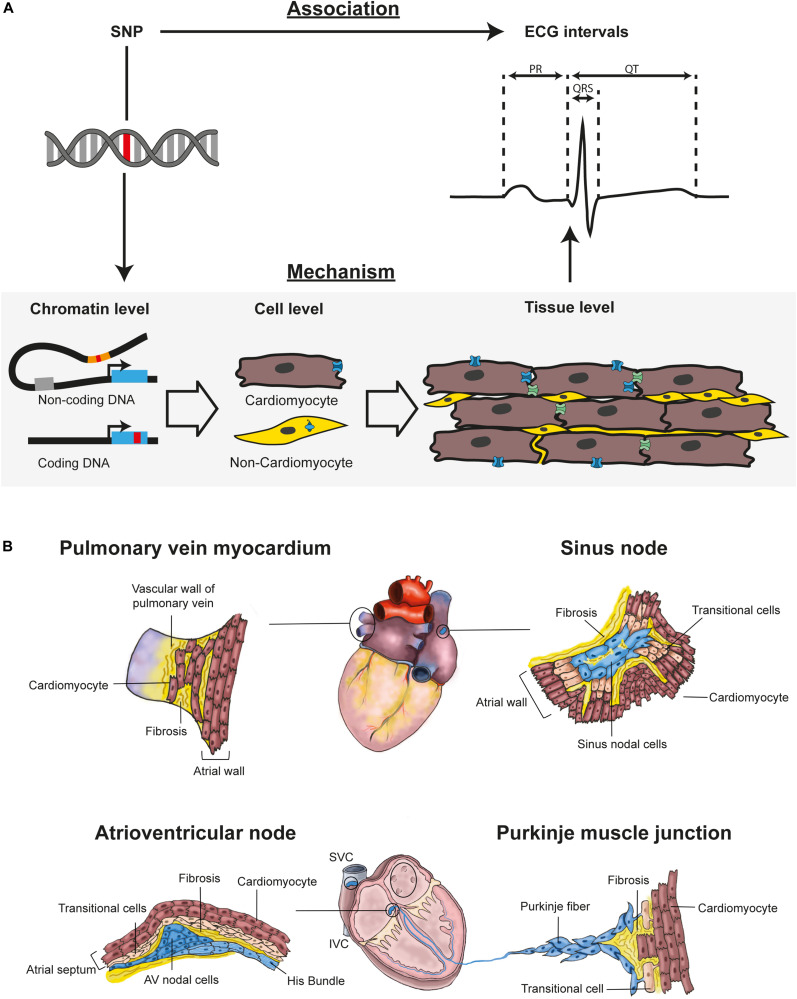
In GWAS, **(A)** SNPs are associated with ECG intervals, like PR interval, QRS complex, and QT interval. To understand physiological relevance of a SNP three biological levels of organization should be studied (1) Chromatin level: Determine which gene(s) is affected by the SNP. The SNP (red mark) can be located in non-coding or coding DNA. For SNPs in non-coding DNA, it is important to determine which coding genes are affected. (2) Cell level: Determine in which cell types the gene is expressed. The cell type in which the affected gene is expressed is relevant for cellular function of the gene. (3) Tissue level: Determine how gene expression in a cell type affects tissue electrophysiology. Tissue electrophysiology can be related to ECG intervals. **(B)** Tissue structure is essential for proper conduction. The pulmonary vein has an outer sleeve of cardiomyocytes. These pulmonary vein cardiomyocytes are not as densely packed as the atrial cardiomyocytes – resulting in less coupling. Small reentry circuits or ectopic activity can therefore occur in pulmonary vein myocardium. The sinus node – where it is not insulated by fibrosis – connects with the atrial cardiomyocytes through transitional cells. In the atrioventricular junction, AV nodal cells are electrically connected with the atrial myocardium through a layer of transitional cells. In the Purkinje muscle junction, Purkinje cells connect with ventricular cardiomyocytes through transitional cells. AV, atrioventricular; IVC, inferior vena cava; SNP, Single nucleotide polymorphism; SVC, superior vena cava.

Genome Wide Association Studies (GWAS) can identify genetic variants associated with phenotypic traits, such as electrocardiographic (ECG) intervals (Table 1). Interpretation of GWAS data relies on identification of the target gene affected by the novel discovered variant. Variants can be present in coding DNA, causing amino acid changes affecting protein function – or in non-coding DNA, altering the behavior of regulatory elements, thereby changing expression levels of its target gene(s) (Figure 1A). For many highly significant GWAS loci the gene causing the association with ECG intervals has not been identified yet. This is mostly because genes in or near these loci do not have a proven association with the phenotype. Overlaying such loci with publicly available genome wide data sets of gene expression (GTEx Consortium, 2013; Heinig, 2018), cardiac transcriptional elements (van Duijvenboden et al., 2016), genomic conformation Hi-C data sets (Montefiori et al., 2018), and other GWAS results will likely provide more insight into potential novel candidate genes regulating cardiac electrophysiology (van Ouwerkerk et al., 2019).

**TABLE 1:** Level of functional studies for GWAS loci associated with ECG intervals.

Associated gene	G	C	T
**AF**			
AGBL4	1		
AKAP6	1		
ARHGAP10	1		
ARHGAP26/NR3C1	1		
ASAH1	1		
ATXN1	1	6	
BEST3	1		
ClOorfll	1		
C10orf76	1		
C20orfl66	1		
C6orfl/NUDT3	1		
C9orf3	1		
CAND2	1		13
CASC20/BMP2	1		14
CASZ1	1		
CDK6	1		
CEP68	1		
CGA/ZNF292	1		
COG5	1		
CREB5	1		
CUL4A	1		
CYTH1	1		
DGKB	1		
DNAH10	1		
DPF3	1		
EPHA3	1		
ERBB4	1	25	26
FBN2/SLC27A6	1		
FBRSL1	1		
FBX032	1		
GCOM1	1		
GJA5	1	32	32
GOPC	1		
GORAB/PRRX1	1	35	
GTF2I	1		
GYPC	1		
HAND2	1		
HIP1R	1		
HSPG2/CELA3B	1		
IGF1R	1	38	39
KCND3	1	40	
KCNJ5	1	45	46
KCNN2	1	47	48
KCNN3/PMVK	1	50	51
KDM1B	1		
KIF3C	1		
KRR1/PHLDA1	1		
LHX3	1		
LINC00208/GATA4	1		
LINC00326/EYA4	1		
LINC00540/BASP1P1	1		
LINC00927/ARNT2	1		
LOC100506385	1		
LOC102467213/EFNA5	1		
LRRC74/IRF2BPL	1		
MAPT	1	61	
MBD5	1		
METTL11B/LINC01142	1		
MEX3C	1		
MIR30B	1		
MTSS1/LINC00964	1		
MY018B	1		
NACA	1		
NEURL	1	13	
NUCKS1	1		
OPN1SW	1		
PAK2	1		
PITX2/C4orf32	1	69	69
PKP2	1	70	
POLR2A/TNFSF12	1		
PPFIA4	1		
PPP2R3A	1		
PRDM8/FGF5	1		
PSMB7	1		
PTK2	1		
RBM20	1	81	
REEP1/KDM3A	1		
REEP3	1		
RPS2	1		
SCMH1	1		
SIRT1	1	2	3
SLC24A2/MLLT3	1		
SLC9B1	1		
SLIT3	1		
SMAD7	1		
SNRNP27	1		
SNX6/CFL2	1		
SORL1/MIR100HG	1		
SSPN	1		
SUN1	1		
SYNE2	1		
SYNP02L	1		
TEX41	1		
THRB	1		
TUBA8	1		
UBE4B	1		
USP3	1		
UST	1		
WDR1	1		15
WIPF1/CHRNA1	1		
WNT8A/NME5	1		
XPOl	1		
XP07	1		
XXYLT1	1		
YWHAE/CRK	1		
ZFHX3	1	20	21
ZNF462	1		
ZPBP2	1		
**PR**			
ALDH18A1/SORBS1	29		
ARHGAP24	29–31		
CCNL1	29		
EFHA1	29		
EOMES	29	34	
EPS15	29		
FAT1	29		
FERMT2	29		
FGFR1	29		
FIGN	29		
ID2	29		37
KRTCAP2	29		
LRCH1	29		
MED13L	29		
MEIS1	29		
MKLN1	29		
MYBPHL	29		52
OBSCN	29		
PAM	29		
PDZRN3	29		
SENP2	29	57	
SH3PXD2A/OBFC1	29		
SKI	29		
SOX5	29	59	60
TMEM182	29		
WNT11	29,31		
XP04	29		
ZFPM2	29		
**HR**			
ACHE	41	63	64
B3GNT7	41		
CD34	41		
CD46	41		
CHRM2	41		
CPNE8	41		
FADS1	41,42		
FLRT2	41		
FNDC3B	41		
RFX4	41		
SLC12A9	41		
TFPI	41		71
UfSpl	41		
**HRV**			
NDUFA11	65		
NEOl	65		
PPIL1	65		
RGS6	65	77	78
**QRS**			
CRIMl	7		
DKK1	7	83	
HAND1/SAP30L	7		
HEATR5B/STRN	7		
IGFBP3	4		
NFIA	4		
SETBP1	4		
TKT/PRKCD/CACNA1D	4	5	5
VTI1A	4		
**QT**			
ANKRD9	7		
ATP1B1	7	8	
ATP2A2	7	9	10
AZIN1	7		
C30RF75	7		
c6orf204	7		
CNOT1	7,11		12
CREBBP	7		
FEN1/FADS2	7		
GBF1	7		
GFRA3	7		
GMPR	7		
KCNE1	7,11	16	17
KCNQ1	7,11	18	19
LAPTM4B	7		
LIG3	7,11		
LITAF	7,11		
MKL2	7		
NCOA2	7		
NOS1AP	7,11	22	23
RNF207	7,11	24	24
SLC4A4	7	27	
SLC8A1	7	28	28
SMARCAD1	7		
SP3	7		
TCEA3	7		
USP50/TRPM7	7	33	
**AF+PR**			
CAMK2D	1,29	36	36
FRMD4B	1,29		
MYOCD	1,29		
NAV2	1,29		
PHLDB2	1,29		
TLE3/UACA	1,29		
**AF+HR**			
GJAl	1,41,42	43	44
GNB4	1,41		
MYH7	1,42	49	
**AF+QT**			
KCNH2	1,7,11	53	54
KCNJ2/CASC17	1,7	55	56
SPATS2L	1,7		
**AF+QRS**			
C1orfl85/RNF11/DKN2C/FAF1	1,4		
CASQ2	1,4	58	58
CDKN1A	1,4,30		
GOSR2	1,4		
LRIG1/SLC25A26	1,4		
**PR+QRS**			
SNORD56B/SIPA1L1	4,29		
TBX20/HERPUD2	4,29	62	
**HR+HRV**			
GNG11	41,65		
KIAA1755	41,65		
SYT10	41,65		
**QRS+QT**			
KLF12	4,7		
PRKCA	4,7		
**HR+HRV+AF**			
HCN4	1,41,65	66	66
**HR+SSS+AF**			
MYH6	29,30,41,42,67	68	
**HR+AF+PR**			
NKX2-5/BNIP1	1,29,31,41	72	72
**AF+PR+OT**			
CAV1/CAV2	1,7,29,31	73	74
**AF+PR+QRS**			
TBX5/TBX3	1,4,29,30,75	76	76
**PR+QRS+QT+AF**			
SCN5A/SCN10A/EXOG	1,4,7,11,29,30,75	79	80
**HR+AF+PR+QT**			
TTN/CCDC141	1,7,29,41		82
**HR+HRV+AF+PR**			
LINC00477/SOX5/BCAT1	1,29,41,42,65	59	60
**HR+AF+QRS+QT**			
C6orf204/SLC35F1/PLN/BRD7P3	1,4,7,41,42	84	84

*Column G: GWAS reference. Column C: reference with electrophysiological experiments in cells. Column T reference with electrophysiological experiments in tissue. AF, atrial fibrillation; PR, PR interval; HR, heart rate; HRV, heart rate variability; QRS, QRS duration; QT, QT interval; SSS, sick sinus syndrome. ^1^Roselli et al., 2018; ^2^Vikram et al., 2017; ^3^Liu et al., 2016; ^4^Sotoodehnia et al., 2010; ^5^Zhang et al., 2005; ^6^Inoue et al., 2001; ^7^Arking et al., 2014; ^8^Barwe et al., 2009; ^9^Suarez et al., 2004; ^10^Shin et al., 2011; ^11^Newton-Cheh et al., 2009; ^12^Yamaguchi et al., 2018; ^13^Sinner et al., 2014; ^14^Howden et al., 2013; ^15^Huang et al., 2019; ^16^Mazhari et al., 2002; ^17^Drici et al., 1998; ^18^Moreno et al., 2015; ^19^Knollmann et al., 2004; ^20^Kao et al., 2016; ^21^Cheng et al., 2019; ^22^Kapoor et al., 2014; ^23^Sugiyama et al., 2016; ^24^Roder et al., 2014; ^25^Yang et al., 2019; ^26^Zhou et al., 2018; ^27^Myers et al., 2016; ^28^Pott et al., 2007; ^29^van Setten et al., 2018; ^30^Holm et al., 2010; ^31^Pfeufer et al., 2010; ^32^Noureldin et al., 2018; ^33^Sah et al., 2013; ^34^Pfeiffer et al., 2018; ^35^Tucker et al., 2017; ^36^Purohit et al., 2013; ^37^Moskowitz et al., 2007; ^38^Wang et al., 2014; ^39^Sapra et al., 2014; ^40^Lugenbiel et al., 2018; ^41^den Hoed et al., 2013; ^42^Eijgelsheim et al., 2010; ^43^Lübkemeier et al., 2013; ^44^Tuomi et al., 2011; ^45^Bender et al., 2001; ^46^Kelly et al., 2014; ^47^Li et al., 2009; ^48^Yi et al., 2015; ^49^Yang et al., 2018; ^50^Zhang et al., 2014; ^51^Mahida et al., 2014; ^52^Barefield et al., 2017; ^53^Orvos et al., 2019; ^54^Liu et al., 2017; ^55^Hattori et al., 2012; ^56^Zaritsky et al., 2001; ^57^Dustrude et al., 2013; ^58^Song et al., 2007; ^59^Zheng et al., 2002; ^60^Li et al., 2013; ^61^Rocher et al., 2010; ^62^Caballero et al., 2017; ^63^Amend et al., 2019; ^64^Masuda and Kawamura, 2003; ^65^Nolte et al., 2017; ^66^DiFrancesco and Borer, 2007; ^67^Holm et al., 2011; ^68^Herron et al., 2010; ^69^Syeda et al., 2016; ^70^Sato et al., 2009; ^71^Goldfarb et al., 1998; ^72^Li et al., 2019; ^73^Barbuti et al., 2012; ^74^Park et al., 2002; ^75^Smith et al., 2011; ^76^Dai et al., 2019; ^77^Posokhova et al., 2010; ^78^Posokhova et al., 2013; ^79^Berecki et al., 2010; ^80^Zhang et al., 2007; ^81^van den Hoogenhof et al., 2018; ^82^Ahlberg et al., 2018; ^83^Eldabah et al., 2018; ^84^Bai et al., 2013.*

Upon discovery, the expression of the target gene is often modified in isolated cardiomyocytes (from an animal model or human induced pluripotent stem cells). These cellular models can be used to assess any electrophysiological phenotypes associated with the potential GWAS candidate. However, ECG intervals reflect the complex electrophysiological interaction of the cardiac cells and tissue structures, complicating extrapolation of single cell measurements to ECG intervals (Opthof et al., 1987). Additionally, discovered variants may affect expression of genes in non-cardiomyocytes, further complicating the interpretation of such experiments in isolated cardiomyocytes (Stroud et al., 2016; Veerman et al., 2016). To overcome these limitations, *in vivo* models such as transgenic mice are most commonly used to investigate the relation between GWAS variants and electrophysiological phenotypes. However, large electrophysiological differences exist between mice and larger mammals such as human (Boukens et al., 2014), which need to be taken into account when interpreting the resulting data.

In this review, we give an overview of genomic loci related to abnormal cardiac electrophysiology and discuss how these variants may directly or indirectly influence ECG intervals and the occurrence of arrhythmias through their expression pattern and regulation within the heart.

## GWAS Loci Related to Heart Rate

The sinus node is located at the border between intercaval area and the right atrium. Action potentials of sinus node cardiomyocytes have low upstroke velocities and occur spontaneously (West, 1955). The mechanism underlying these spontaneous depolarizations is based on the funny current, mediated by the Hyperpolarization-Activated Cyclic Nucleotide-gated channel 4 (*HCN4*), and the calcium clock mediated by e.g., Ryanodine Receptor 2 (*RYR2*) and Phospholamban (*PLN*) (Lakatta and DiFrancesco, 2009). Accordingly, associations between the genetic loci of *HCN4* and *PLN* and heart rate found by GWAS is most likely mediated through variants impacting on the expression and or function of these genes (Eijgelsheim et al., 2010; den Hoed et al., 2013; Nolte et al., 2017). Unfortunately, for the other GWAS loci, the association is not as straightforward. In the proximity of these loci, no genes with a known function in the SAN have been identified. These loci therefore potentially affect SAN function by impacting on other parameters, e.g., electrical coupling with the rest of the atrium and levels of fibrosis (Glukhov et al., 2013).

In smaller mammals – e.g., mice and rabbits – the sinus node is transmural (Bleeker et al., 1980; van Eif et al., 2019), whereas in large mammals – e.g., humans and dogs – it is not (Fedorov et al., 2009, 2010). The geometry of the sinus node affects coupling with atrial myocardium, which influences its electrophysiological behavior (Kirchhof et al., 1987). The delicate interaction between the sinus node and the surrounding atrial myocardium is established by fibrous tissue providing up to five exit pathways in large mammals (Figure 1B; Unudurthi et al., 2014; Csepe et al., 2015; Li et al., 2017). A transitional layer of cells within the exit pathways (Figure 1B) allows for spontaneous depolarization within the sinus node (Joyner and van Capelle, 1986; Kléber and Rudy, 2004). The identity of the transitional cells may depend on the expression of transcription factor Nkx2-5 and GWAS has found variants near NKX2-5 associating with heart rate (Mommersteeg et al., 2007b; den Hoed et al., 2013; Li et al., 2019). Within the exit pathways, high conductance connexins CX43 and CX40 (*GJA1* and *GJA5*) are lower expressed toward the sinus node, whereas the low conductance CX45 is expressed higher toward the sinus node (Chandler et al., 2009, 2011; Allah et al., 2011). This gradual increase of conductance from sinus node toward atrial myocardium enables a proper current-to-load match. Therefore, genes involved in sinus node insulation and exit pathway formation are candidates for being associated with sinus node function (or heart rate) in GWAS. Accordingly, a locus near *GJA1* is associated with heart rate in GWAS (den Hoed et al., 2013).

The electrotonic influence of atrial myocardium on sinus node function is important to consider when investigating the relevance of genes provided by GWAS. For instance, common and rare variants in *MYH6* – a component of the sarcomere – are associated with heart rate and sick sinus syndrome (Holm et al., 2010, 2011). However, *MYH6* is more abundantly expressed in the atrial myocardium than in the sinus node (van Eif et al., 2019). *In vitro* experiments in atrial-like cells indicate that mutations in *MYH6* affect conduction velocity (Ishikawa et al., 2015). It is possible that conduction slowing in the atrium underlies the association between *MYH6* and abnormal function of the sinus node. That genes expressed in the atrium but not in the sinus node can affect sinus is further illustrated by Cx40 knock-out mice in which the dominant pacemaker is not always the SAN (Bagwe et al., 2005). Moreover, mutations in *SCN5A* can result in sick sinus syndrome (Benson et al., 2003) despite the lack of *SCN5A* expression in the sinus node.

## GWAS Loci Related to PR Interval

The PR interval is mainly determined by conduction through the atrioventricular junction (Meijler and Janse, 1988). The atrioventricular junction is a complex anatomical structure which was first described in 1906 by Sunao Tawara, who found cells forming a compact complex network but also small cells that joined into bundles (Tawara, 1906). These cells were later called compact nodal cells and transitional cells, respectively, and both have distinct electrophysiological properties (de Carvalho and de Almeida, 1960; Greener et al., 2011). A layer of transitional cells can be found in rings around the orifices of the AV valves where expression of Cx40 and SCN5A is low (Aanhaanen et al., 2010; Fedorov et al., 2011). This transitional ring lies on top of an atrioventricular nodal ring providing two conducting pathways with different electrophysiological characteristics both connecting the atria with the His bundle (Denes et al., 1973; Figure 1B). The presence of two conduction pathways illustrates the challenge of relating gene expression of single atrioventricular nodal cells to the electrophysiological phenotype of the atrioventricular node. Moreover, in the atrioventricular junction, not only the compact atrioventricular node contributes to atrioventricular delay but also all other cells present within the AV junction – e.g., transitional cells, fibroblasts and macrophages (Hulsmans et al., 2017). Similar to the sinus node, the function of the atrioventricular node depends on the interaction with these different cell-types.

The cells of the atrioventricular junction find their origin in the atrioventricular canal of the embryonic heart. Impulse conduction delay occurs in both the transitional cells and the compact atrioventricular node, therefore, normal development of the atrioventricular canal is crucial for atrioventricular delay in adult hearts (Meijler and Janse, 1988). Accordingly, GWAS for PR interval showed association with 18 of 44 loci which are related to heart development (van Setten et al., 2018), indicating that proper embryonic development is a crucial factor for adult AV conduction. Some of these loci are located near *TBX2* and *TBX3* – essential transcription factors controlling patterning of the atrioventricular canal during development (Holm et al., 2010; Pfeufer et al., 2010; Aanhaanen et al., 2011; van Setten et al., 2018; Table 1). Other loci are located nearby genes related to electrical function of the adult cardiomyocyte, like *SCN5A* and *CAMK2D* or cardiomyocyte contraction, like *TTN* and *MYH6*, however, the significance of these associations with PR interval await further investigation (Holm et al., 2010; van Setten et al., 2018).

## GWAS Loci Related to QRS Duration

Total ventricular activation time is visualized on the ECG as the duration of the QRS complex, which comprises conduction in the His-Purkinje system and in the ventricular myocardium. The cardiac sodium channel Na_v_1.5 – encoded by *SCN5A* – is the major determinant of conduction in these tissues and, accordingly, QRS duration is associated with loci near *SCN5A* (Papadatos et al., 2002; Sotoodehnia et al., 2010). Variants in the *SCN10A* gene – encoding the neuronal sodium channel Na_v_1.8 – associate with QRS duration as well (Sotoodehnia et al., 2010). These variants are located within an enhancer region that modulates expression of *SCN5A* which could explain a relation with QRS duration (Sotoodehnia et al., 2010; van den Boogaard et al., 2014). Although variants in these region do not always associate with QRS prolongation (Behr et al., 2015). Other variants related to QRS duration are those near or within genes involved in bundle branch development and working myocardial phenotype, like *TBX3*, *TBX5*, *TBX20*, *HAND1*, *DKK1*, and *NFIA* (Moskowitz et al., 2007; Bakker et al., 2008; Singh et al., 2009; Sotoodehnia et al., 2010). In addition variants in calcium handling genes such as *PLN*, *CACNA1D*, *STRN*, *PRKCA*, and *CASQ2, ATP2A2/ANAPC7* are associated with QRS duration (Sotoodehnia et al., 2010; van Setten et al., 2019). These variants could affect calcium homeostasis resulting in reduced sodium current and slow conduction and thereby prolong QRS duration (King et al., 2013).

The Purkinje network activates the ventricular myocardium via Purkinje muscle junctions composed of transitional cells connecting the Purkinje fibers to ventricular cardiomyocytes (Figure 1B; Martinez-Palomo et al., 1970; Tranum-Jensen et al., 1991). Similar to the AV node and the SAN, the connection of Purkinje cardiomyocytes with ventricular cardiomyocytes requires high resistance – preventing current-to-load mismatch (Rohr et al., 1997). We expect that reduction of electrical coupling in these junctions – by e.g., lower expression of CX40 – will delay ventricular activation, which prolongs QRS duration. Purkinje fibers develop from embryonic trabeculae (Jensen et al., 2012) where e.g., Cx40, *Scn5a*, *CnTn2* are abundantly present. The primordial Purkinje trabeculae require further specialization after birth under influence of *Nkx2*-5 and *Irx3* expression and Notch signaling (Zhang et al., 2011; Rentschler et al., 2012). Homozygous *Irx3* loss-of-function mice have slowed conduction in Purkinje fibers (Koizumi et al., 2016). We expect that genetic variations affecting expression of these factors – that are involved in development and maturation of the Purkinje fibers – will relate to total ventricular activation time and thereby QRS duration in GWAS.

## GWAS Loci Related to QT Interval

The QT interval is a measure of ventricular repolarization and reflects the time between the first moment of activation to the last moment of repolarization. Accordingly, the QT interval relates to action potential duration (APD) but also to differences in regional conduction velocity. Conduction slowing in regions that repolarize late prolongs QT interval whereas conduction slowing in areas that repolarize early may not affect QT interval. The QT interval has an inverse relation with heart rate. This inverse relation results from shortening of the APD at higher rates due to activation of the slowly delayed rectifier current I_Ks_ (Boyett and Jewell, 1978; Carmeliet, 2006). QT intervals corrected for heart rate – QTc – or QT interval measured at similar heart rates may increase sensitivity for finding variants related to repolarization (Bazett, 1920). In mice, however, QT interval does not depend on heart rate. Therefore, QT interval – not QTc – should be used as measure for ventricular repolarization in mice (Speerschneider and Thomsen, 2013).

Genome Wide Association Studies for QT interval identified 22 loci (Table 1) of which many are in or near genes encoding for potassium channels or involved in calcium handling (Arking et al., 2014). Increased potassium current shortens QT interval whereas increased calcium current prolongs QT interval (Wemhöner et al., 2015; Landstrom et al., 2016). Nitric Oxide Synthase 1 Adaptor Protein (*NOS1AP*) – regulating calcium current – is associated with QT interval in GWAS (Arking et al., 2006, 2014). Genetic variation within the *NOS1AP* gene affects QT interval and is related to arrhythmogenesis in patients with the long QT syndrome (Crotti et al., 2009; Tomás et al., 2010). The effect of *NOS1AP* on QT interval, however, could also have an extracardiac pathway as its expression is high in brain (Xu et al., 2005) providing the possibility of *NOS1AP* to affect autonomic modulation of the QT interval (Yagishita et al., 2015).

## GWAS Loci Related to Arrhythmias

### Atrial Arrhythmias

Atrial fibrillation (AF) is the most common arrhythmia and the prevalence increases with age (Krijthe et al., 2013). AF results from an interplay between electrical (triggers and reentry), structural and hemodynamic remodeling (Schotten et al., 2011). The combination of these pathophysiological changes sets the stage for AF.

The trigger for AF is commonly near the connection between the left atrium and pulmonary veins (PVs) (Haïssaguerre et al., 1998). In humans – at this connection – the four PVs are enclosed by an outer sleeve of myocardium (Saito et al., 2000). PV cardiomyocytes are morphologically similar to atrial cardiomyocytes, but have a different developmental history (Verheule et al., 2002; Mommersteeg et al., 2007a). The formation of PV myocardium in mice highly depends on expression of *Pitx2* and *Nkx2-5* during development (Mommersteeg et al., 2007a). GWAS in more than 65 thousand AF patients identified 97 loci (Roselli et al., 2018) – including *PITX2* and *NKX2-5*, suggesting a relation of AF with PV formation during development. The myocardial sleeves of the PVs are thinner distal to the left atrium and end in single cardiomyocyte protrusions in the PV (Figure 1B; Verheule et al., 2002). These PV myocytes have a high resting membrane potential and low expression of inward rectifier channels (Ehrlich et al., 2003; Melnyk et al., 2005), setting the stage for spontaneous activity. Accordingly, variants near genes encoding inward rectifier channels – e.g., *KCNJ2, KCNJ5* – are associated with AF (Christophersen et al., 2017; Roselli et al., 2018).

Electrical remodeling is a cause and effect of reentry circuits that maintain AF. Reentry is facilitated by slow conduction and short APD (Wiener and Rosenblueth, 1946). Accordingly, GWAS for AF identified six genes encoding for potassium channels all involved in atrial APD: *KCND3*, *KCNH2*, *KCNN2*, and *KCNN3* (Roselli et al., 2018). Genes encoding channels involved in conduction – e.g., *GJA1*, *GJA5*, *SCN5A*, and *SCN10A* are also associated with AF (Christophersen et al., 2017; Roselli et al., 2018). Moreover, transcription factors involved in spatiotemporal expression of these ion channels – *TBX3*, *TBX5*, and *PITX2* – associate with AF as well (Tao et al., 2014; Nadadur et al., 2016). Novel findings have indicated that not only genes encoding ion channel are related to AF. Titin (TTN) – a large sarcomere protein – is associated with early onset AF (Choi et al., 2018). TTN dysfunction may predispose to AF by increasing myocardial fibrosis and prolonging PR interval, which are both associated with increased risk for AF (Ahlberg et al., 2018).

### Ventricular Arrhythmias

Up to 80% of sudden cardiac arrests (SCA) are caused by acute ischemia resulting from coronary artery disease (Huikuri et al., 2001; Fishman et al., 2010). GWAS in patients with coronary artery disease identified 11 variants related to SCA of which eight were near or within genes related to long QT syndrome (Marsman et al., 2014). It is unclear whether these variants predispose to arrhythmias in general or only in the setting of coronary artery disease. A gene that does specifically relate to arrhythmias in the setting of acute myocardial ischemia is *CXADR* which encodes the CXADR Ig-like cell adhesion molecule (previously named Coxsackie and adenovirus receptor, CAR) (Bezzina et al., 2010). Reduced expression of CAR lowers sodium channel availability – thereby reducing conduction velocity – and facilitates reentry arrhythmias in the setting of ischemia (Marsman et al., 2014). Non-ischemia induced arrhythmias explain 20% of SCA and comprise a variety of arrhythmogenic syndromes which can be related to structural abnormal myocardium or genetic mutations (John et al., 2012). GWAS for SCA identified several genes which all associate with risk factors for SCA such as QRS duration and QT interval (Adabag et al., 2010; Arking et al., 2011; Milano et al., 2016; Ashar et al., 2018).

Genome Wide Association Studies for syncope – which is a common symptom of many arrhythmogenic syndromes – identified a genetic variant close in proximity to the gene zinc finger protein 804a (*ZNF804A*) (Hadji-Turdeghal et al., 2020). Whether a role of *ZNF804A* in cardiac arrhythmias or e.g., blood pressure regulation explains this association remains to be investigated.

Ventricular fibrillation is associated with IRX3 (Koizumi et al., 2016), which plays a role in conduction in Purkinje fibers. Ablation of Purkinje muscle junctions is a successful treatment for a subset of patients with idiopathic ventricular fibrillation indicating a role of Purkinje cells in these arrhythmias (Haïssaguerre et al., 2002).

Understanding arrhythmogenic mechanisms can guide interpretation of GWAS derived data. This is exemplified by the Brugada syndrome, which is characterized by ST segment elevation in the right precordial leads and ventricular arrhythmias. Initially, the Brugada syndrome was considered an ion channel disease mainly resulting from dysfunction of the cardiac sodium channel. However, only 20% of patients present with mutations in *SCN5A* (Antzelevitch et al., 2005). A GWAS in Brugada syndrome patients identified several variants in regulatory DNA controlling SCN5A expression (Bezzina et al., 2013). In order to discover causal variants in GWAS with limited number of patients, knowledge on the arrhythmogenic mechanism of the disease is helpful. Mechanistic studies in hearts from Brugada syndrome patients have suggested that arrhythmias occur due to conduction block in the presence of subtle structural abnormalities (Coronel et al., 2005; Hoogendijk et al., 2010). This suggests that variants near genes involved in the formation of fibrosis (*TGFB2)* or genes affecting safety factor of cardiac conduction (e.g., *SCN5A*, *GJA1*, *KCHIP1*) are important variants to further investigate.

## Conclusion

Most of the variants provided by GWAS lie near or within expected candidate genes potentially explaining the phenotype they are associated with. However, the electrophysiological characterization of the vast majority of associated genes has not shown direct effects on ECG intervals nor on arrhythmia susceptibility. In this review, we emphasize that mechanistic knowledge of the structure-function relations underlying ECG intervals and arrhythmias should be considered when interpreting experimental characterization of these variants, in order to guide clinical applicability of GWAS data.

## Author Contributions

KS and BB designed and wrote the manuscript. VM, CG-M, and EL critically revised the manuscript.

## Conflict of Interest

The authors declare that the research was conducted in the absence of any commercial or financial relationships that could be construed as a potential conflict of interest.
